# Granulocytes-Rich Thrombi in Cerebral Large Vessel Occlusion Are Associated with Increased Stiffness and Poorer Revascularization Outcomes

**DOI:** 10.1007/s13311-023-01385-1

**Published:** 2023-05-22

**Authors:** Jesús Juega, Jiahui Li, Carlos Palacio-Garcia, Maite Rodriguez, Riccardo Tiberi, Carlos Piñana, David Rodriguez-Luna, Manuel Requena, Álvaro García-Tornel, Noelia Rodriguez-Villatoro, Marta Rubiera, Marian Muchada, Marta Olivé-Gadea, Federica Rizzo, David Hernandez, Marta Dios-Lascuevas, Maria Hernandez-Perez, Laura Dorado, Helena Quesada, Pere Cardona, Carolina De La Torre, Laura Gallur, Jessica Camacho, Santiago Ramon-y-Cajal, Alejandro Tomasello, Marc Ribó, Carlos A. Molina, Jorge Pagola, the ITACAT study group

**Affiliations:** 1 Stroke Unit, Department of Neurology, Vall d’Hebron University Hospital, Vall d’Hebron Research Institute. Universitat Autonoma de Barcelona, Passeig de la Vall d’Hebron, 119-129, Barcelona, 08035 Spain; 2 Hematology Department, Vall d’Hebron University Hospital, Barcelona, Spain; 3 Department of Neuroradiology, Vall d’Hebron University Hospital, Barcelona, Spain; 4 Department of Neurology, Germans Trias I Pujol University Hospital, Badalona, Spain; 5 Department of Neurology, Bellvitge University Hospital, Hospitalet de Llobregat, Spain; 6 Proteomics Unit, Josep Carreras Leukaemia Research Institute (IJC), Badalona, Spain; 7 Department of Pathology, Vall d’Hebron University Hospital, Barcelona, Spain

**Keywords:** Acute stroke, Mechanical thrombectomy, Flow cytometry, Granulocytes, Recanalization, Thrombi

## Abstract

We aim to identify a profile of intracranial thrombus resistant to recanalization by mechanical thrombectomy (MT) in acute stroke treatment. The first extracted clot of each MT was analyzed by flow cytometry obtaining the composition of the main leukocyte populations: granulocytes, monocytes, and lymphocytes. Demographics, reperfusion treatment, and grade of recanalization were registered. MT failure (MTF) was defined as final thrombolysis in cerebral infarction score IIa or lower and/or need of permanent intracranial stenting as a rescue therapy. To explore the relationship between stiffness of intracranial clots and cellular composition, unconfined compression tests were performed in other cohorts of cases. Thrombi obtained in 225 patients were analyzed. MTF were observed in 30 cases (13%). MTF was associated with atherosclerosis etiology (33.3% vs. 15.9%; *p* = 0.021) and higher number of passes (3 vs. 2; *p* < 0.001). Clot analysis of MTF showed higher percentage of granulocytes [82.46 vs. 68.90% *p* < 0.001] and lower percentage of monocytes [9.18% vs.17.34%, *p* < 0.001] in comparison to successful MT cases. The proportion of clot granulocytes (aOR 1.07; 95% CI 1.01–1.14) remained an independent marker of MTF. Among thirty-eight clots mechanically tested, there was a positive correlation between granulocyte proportion and thrombi stiffness (Pearson’s *r* = 0.35, *p* = 0.032), with a median clot stiffness of 30.2 (IQR, 18.9–42.7) kPa. Granulocytes-rich thrombi are harder to capture by mechanical thrombectomy due to increased stiffness, so a proportion of intracranial granulocytes might be useful to guide personalized endovascular procedures in acute stroke treatment.

**Supplementary Information:**

The online version contains supplementary material available at 10.1007/s13311-023-01385-1.

## Introduction

Achieving complete recanalization in first attempt is the main goal of mechanical thrombectomy (MT) among patients with an acute stroke due to a large vessel occlusion (LVO). However, patients who achieve successful reperfusion after several passes have better clinical outcomes than those without any reperfusion. Therefore, final revascularization beyond the first pass is still the goal of MT [1–4].

The rate of MT failure in LVO strokes ranges from 10 to 30%; these cases are associated with very poor prognosis [5]. Main causes of MT failure are difficulty to access to target occlusion or failure to retrieve the clot despite multiple attempts [6, 7].

The angiographic outcome of MT procedures is highly influenced by the composition and mechanical properties of the intracranial thrombi. Previous work revealed that erythrocyte-rich thrombi were associated with shorter arrival-to-recanalization time intervals and more favorable clinical outcomes [8]. Moreover, recent studies have shown that neutrophil extracellular traps (NETS) contribute to resistance to reperfusion therapy with more number of passes and less favorable recanalization [9].

Stent retriever and aspiration techniques are the first-line therapy in endovascular treatment, but complex MT procedures may need to set a permanent intracranial stenting (PIS) as a rescue technique after failed standard MT, even though PIS requires strong antithrombotic therapy [5, 10].

The development of markers to predict failure of standard MT may select patients for early angioplasty and PIS in the acute phase of stroke in order to avoid complications associated with multiple attempts. The aim of our study was to define a profile of thrombectomy-resistant thrombus by mechanical characterization and relationship with composition by flow cytometry analysis of retrieved thrombi.

## Methods

### Study Setting and Population

We conducted a prospective, single-center observational study of patients with acute ischemic stroke and LVO who underwent MT from January 2017 to January 2021 to analyze predictors of MT failure and another observational study between March 2021 and June 2021 to perform mechanical characterization with samples from retrieved clots. These studies were included in the multi-center ongoing registry ITACAT, “Impact of Thrombus Analysis in Stroke Patients in Catalonia” (355/C/2017). Demographic data, vascular risk factors, **s**everity of stroke prior to MT as assessed by the National Institutes of Health Stroke Scale (NIHSS), cerebral infarct characteristics on baseline computerized tomography scan, angiographic features, and reperfusion treatment modality in each patient were collected. The etiology of the stroke was classified according to TOAST classification [11]. The study was approved by the Local Committee of Ethics in Medical Research of the Hospital Universitari Vall d’Hebron (PR (AG) 234/2017) and was carried out in line with the second Declaration of Helsinki. Written informed consent was obtained from all participants or their relatives.

### Neuroimaging and Thrombectomy Procedure

According to stroke code protocol, every patient underwent plain CT at the emergency room or at the angio suite in order to rule out intracranial hemorrhage [12]. ASPECTS scores were recorded for each patient when standard CT scan was performed. Reperfusion treatment with intravenous alteplase (iv-rTPA) and/or MT was administered following current guidelines recommendations [13]. Intracranial and/or extracranial LVO was recorded from the diagnostic angiography. All the images were reviewed by an expert radiologist blinded to clinical data. LVOs occlusion were categorized into two groups: proximal large vessel occlusions (PLVOs) in case of occlusion of intracranial and/or extracranial carotid artery; segment M1 of middle cerebral artery (M1-MCA) and basilar artery occlusion (BAO); medium-vessel occlusions (MeVOs) in case of occlusion of distal medium arterial arbors (anterior cerebral artery [ACA], M2 middle cerebral artery [M2-MCA] or posterior cerebral artery [PCA] [14, 15]. The device employed to perform the first attempt in MT procedure were categorized into: direct aspiration first pass technique (ADAPT), stent retriever, or combination of both (combined technique) [16].

Additional procedures such as intracranial angioplasty and/or intracranial stenting were registered [5]. Number of passes and modified thrombolysis in cerebral infarction (mTICI) score after MT were documented [17].

MT failure was defined as mTICI score IIa or lower (mTICI ≤ IIa) and/or need of intracranial stenting after conventional MT procedure. Successful MT was categorized as mTICI 2b or higher (final mTICI ≥ 2b).

### Clot Analysis by Flow Cytometry Protocol

The thrombi obtained after MT were suspended in a container with RPMI 1640 medium 1640 (Gibco™) and stored at 4 to 6 °C for no longer than 24 h until analysis was started in order to avoid decrease in detection of cell surface markers from selected cell populations with different half-life time [18, 19]. Leukocytes were obtained and analyzed as previously published [20]. Briefly, the clots were finely minced and disaggregated to obtain a single cell suspension in 2 to 3 mL of RPMI 1640 using a scalpel. Then, cell suspensions were filtered and washed with phosphate-buffered saline (PBS). Leukocytes were pelleted by centrifugation, resuspended in 100 μL of PBS, and labeled by direct immunofluorescence using CD45 monoclonal antibody conjugated with Krome Orange (Beckman Coulter™). Labeled cells were acquired in a Navios EX™ flow cytometer and were analyzed using Kaluza software (Beckman Coulter™). Finally, leukocytes were classified as granulocytes, monocytes, or lymphocytes according to their side scatter complexity and CD45 level of expression. A threshold of at least 100 leukocytes was set for viable clots. In case there were several thrombi extracted from the same or different vessels, clot obtained in the first pass was selected for analysis from each MT.

### Mechanical Characterization

Retrieved clot fragments were stored in Gibco RPMI 1640 medium (Thermo Fisher, Waltham, MA) at 4–6 °C. Mechanical experiments were performed within 48 h from the end of the MT procedure.

Unconfined compression tests were performed to explore the stiffness of the clots [21–23]. Briefly, test specimens are submitted under gradual compression between two parallel rigid plates, and the force loads applied to cause a specific deformation are registered in a stress–strain curve. The experiments were conducted with a Zwick-Line Z0.5TN testing machine equipped with a 5N load cell (Zwick-Roell, Ulm, Germany) (Online Resource 1). In preparation for the mechanical characterization, clot fragments were trimmed to a height between 1 and 2 mm, and test specimens’ diameters (> 1 mm) were measured with a caliper with a resolution of 0.1 mm. Test parameters were configured to conduct non-destructive experiments, so the clot samples can be preserved for the subsequent analysis by flow cytometry. Test samples were pre-conditioned at 37 °C before conducting the experiments. Unconfined compression tests were configured with a preload force of 0.001 N, followed by a gradual compression up to 50% strain at a rate of 0.1 mm/s. The secant modulus, defined as the slope of a line drawn from the origin of the stress–strain diagram and intersecting the curve at the point of interest (0–45% strain), was used to determine the thrombus stiffness.

### Statistical Analysis

Categorical variables were presented as absolute number and percentage in each group (*n*, %). The distribution of continuous variables was assessed by Kolmogorov–Smirnov test. Continuous variables were expressed as medians with interquartile range [IQR]. Relative proportion of each leukocyte population was expressed as percentage (%) of the type of cell in the total leukocyte population. *P*-value below 0.05 was settled for determining significance in two-tailed test result. For categorical variables, statistical significance was assessed by chi-square *r* or Fisher’s exact test. For continuous variables, Mann–Whitney U test was employed. Univariate analysis of demographics, imaging data, MT procedure, and clot analysis were performed to define predictors associated with MT failure. Among MT with multiple passes, receiver operating characteristic (ROC) curve was drawn to estimate the best cut-off point based on Youden index for proportion of granulocytes to predict MT failure. To estimate adjusted odds ratio (aOR) of MTF, predictive models were created using binary logistic regression analysis and adjusted analysis, adjusted by age, sex, distal intracranial occlusions (MeVOs), and significant variables obtained in the univariate model. The relationship between thrombi composition and thrombus stiffness was statistically assessed with 95% confidence intervals. Bivariate correlation tests were conducted to find the Pearson’s coefficient between continuous variables. Statistical analysis was performed by SPSS 21.0^®^ software.

## Results

**Fig. 1:**
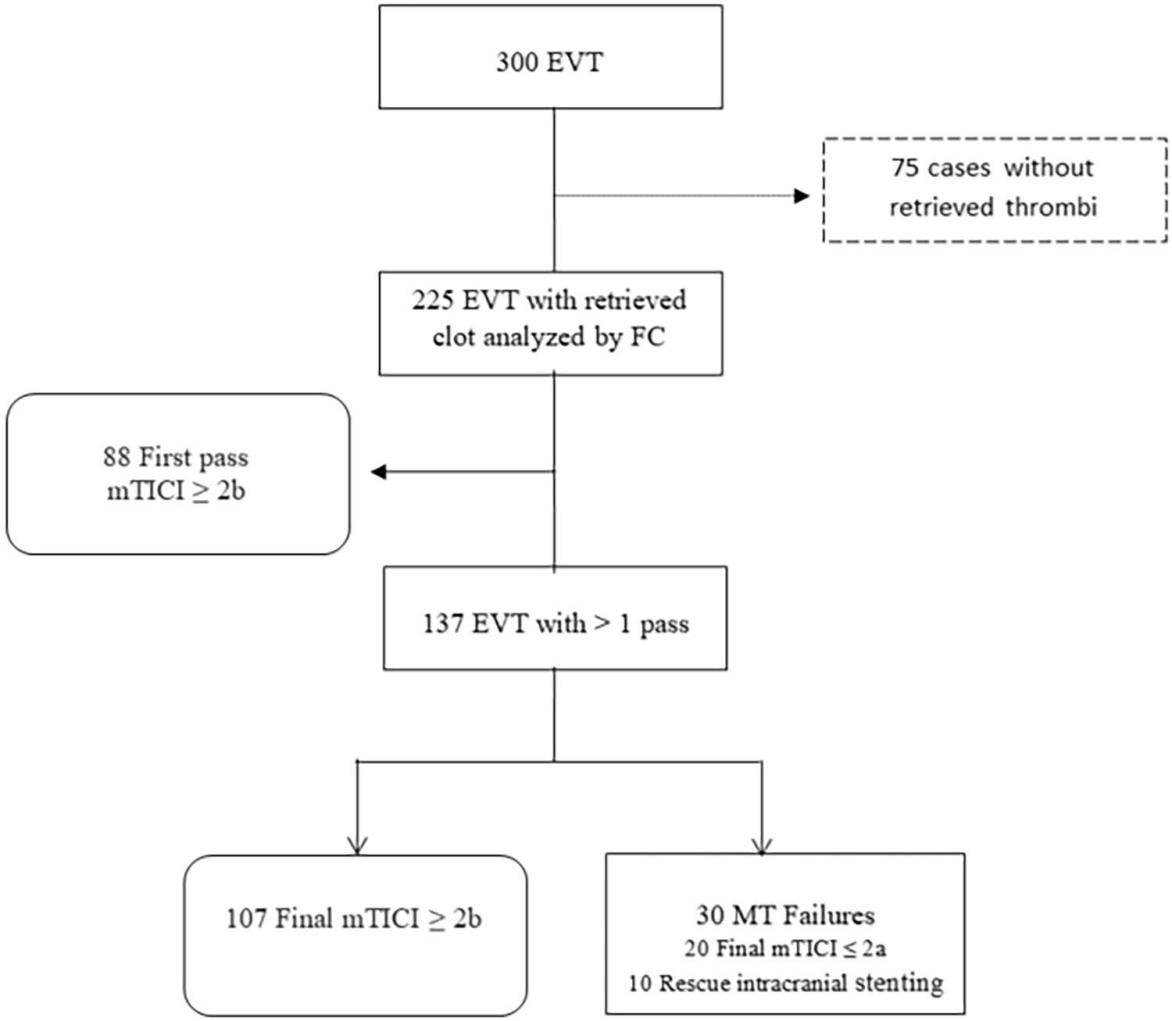
Flow chart. EVT is endovascular treatment; FC is flow cytometry; MT is mechanical thrombectomy; *Mtici* is modified thrombolysis in cerebral infarction; *PIS* is permanent intracranial stenting

From three hundred acute stroke treatments performed from January 2017 to January 2021, two hundred twenty-five retrieved clots were obtained. Thirty-nine percent (88/225) of cases achieved first-pass successful MT and MTF was observed in 30 cases (13% of total MT). Therefore, among 137 endovascular treatments (EVT) that required several passes, 78% (107/137) achieved final mTICI ≥ 2b with successful MT, and 21.89% (30/137) were categorized as MT failure (Fig. 1).

Global data from comparison between MTF versus successful MT (final mTICI ≥ 2b) are stated at Table 1. So therefore, patients with MT failure were more frequently observed in large arterial atherosclerosis stroke [33.3% (10) vs. 15.9% (31); *p* = 0.021] and in MT that required a higher number of passes (3 passes vs. 2; *p* < 0.001). Clot analysis of MT failure showed higher percentage of granulocytes [82.46 (69.19–89.02) vs. 68.90% (58.60–79.07); *p* < 0.001] and lower percentage of monocytes [9.18% (5.29–20.23) vs.17.34% (11.90–27.06); *p* < 0.001]. Neither the use of previous iv-rTPA nor type of device used in first pass was associated with MT failure, as stated in Table 1. Conversely, MT recanalization was associated with high-risk cardioembolic etiology (HRCE) [60% (117) vs. 36.7% (11); *p* = 0.016] and dyslipidemia [52.3% (102) vs. 26.7% (8); *p* = 0.009].

**Table 1:** Global comparison between successful recanalization versus MT failure

	All EVT (*n* = 225)	Successful MT (*n* = 195)	MT failure (*n* = 30)	*P*-value
Age, years [IQR]	75 [63–82]	75 [63–82]	77 [63–83]	0.762
Sex (female) *n*, %	105 (46.7)	88 (45.1)	17 (56.7)	0.238
History of atrial fibrillation *n*, %	65 (28.9)	56 (28.7)	9 (30)	0.885
Active smoker *n*, %	44 (19.6)	37 (19.0)	7 (23.3)	0.575
High blood pressure *n*, %	149 (66.2)	129 (66.2)	20 (66.7)	0.956
Diabetes mellitus *n*, %	43 (19.1)	40 (20.5)	3 (10)	0.173
History of coronary artery disease *n*, %	43 (19.1)	40 (10.6)	3 (10)	0.173
Dyslipidemia *n*, %	110 (48,9)	102 (5.3)	8 (26.7)	0.009
Antiplatelets *n*, (%)	67 (29.8)	59 (30.3)	8 (26.7)	0.689
Anticoagulation *n*, (%)	35 (15.6)	28 (14.4)	7 (23.3)	0.207
Glycemia (mg/dL)	119 [101–137]	120 [102–141]	112 [97–126]	0.206
NIHSS	16 [10–21]	16 [10–21]	16 [8–20]	0.642
HRCE *n*, (%)	128 (56.9)	117 (60)	11 (36.7)	0.016
LAA *n*, (%)	41 (18.2)	31 (15.9)	10 (33.3)	0.021
Other cause *n*, (%)	9 (4.0)	8 (4.1)	1 (3.3)	0.841
ASPECTS score [IQR]	9 [8–10]	10 [8–10]	9 [7–10]	0.254
PLVOs	169 (75.1)	146 (74.8)	23 (76.6)	0.833
TICA occlusion *n*, (%)	52 (23.1)	43 (22)	9 (30)	0.337
Medial artery 1 occlusion *n*, (%)	85 (37.7)	72 (36.9)	13 (43.3)	0.501
Medial artery 1 tandem occlusion *n*, (%)	20 (8.8)	20 (10.3)	0	0.067
BAO occlusion *n*, (%)	12 (5.3)	11 (5.6)	1 (3.3)	0.601
MeVOs	56 (24.8)	49 (25.1)	7 (23.3)	0.833
Anterior artery occlusion *n*, (%)	3 (1.3)	2 (1.05)	1 (3.3)	0.306
Medial artery 2 occlusion *n*, (%)	44 (19.5)	40 (20.5)	4 (13.3)	0.357
Posterior artery occlusion *n*, (%)	9 (0.04)	7 (3.6)	2 (6.7)	0.424
iv–rTPA *n* (%)	78 (34.8)	71 (36.6)	7 (23.3)	0.156
Stent retriever first pass, *n* (%)	63 (28.1)	57 (29.4)	6 (20.0)	0.288
Combined, first pass *n* (%)	130 (0.303)	110 (56.7)	20 (66.7)	0.303
ADAPT first pass *n* (%)	30 (13.4)	27 (13.9)	3 (10.0)	0.558
Number of passes, [IQR]	2 [1–3]	2 [1–3]	3 [2–5]	< 0.001
Clot total leukocytes analyzed [IQR]	3394 [1382–11,593]	3374 [1410–11,641]	3679 [1126–11,188]	0.756
Clot granulocytes % [IQR]	70.31 [59.57–80.68]	68.90 [58.60–79.07]	82.46 [69.19–89.02]	< 0.001
Clot monocytes % [IQR]	16.35 [10.84–25.94]	17.34 [11.90–27.06]	9.18 [5.29–20.23]	< 0.001
Clot lymphocytes % [IQR]	6.65 [2.82–12.90]	8.86 [2.75–13.0]	5.08 [3.0–12.33]	0.340

*ADAPT* a direct aspiration first pass technique, *EVT* endovascular treatment, *HRCE* high risk cardioembolic etiology, *IQR* interquartile range, *Iv-rTPA* intravenous fibrinolysis, *LAA* large arterial atherosclerosis, *MT* mechanical thrombectomy, *NIHSS* National Institutes of Health Stroke Scale, *PLVOs* proximal large vessel occlusions, *MeVOs* medium vessel occlusions

### Comparison Among EVT with Multiple Passes

**Fig. 2:**
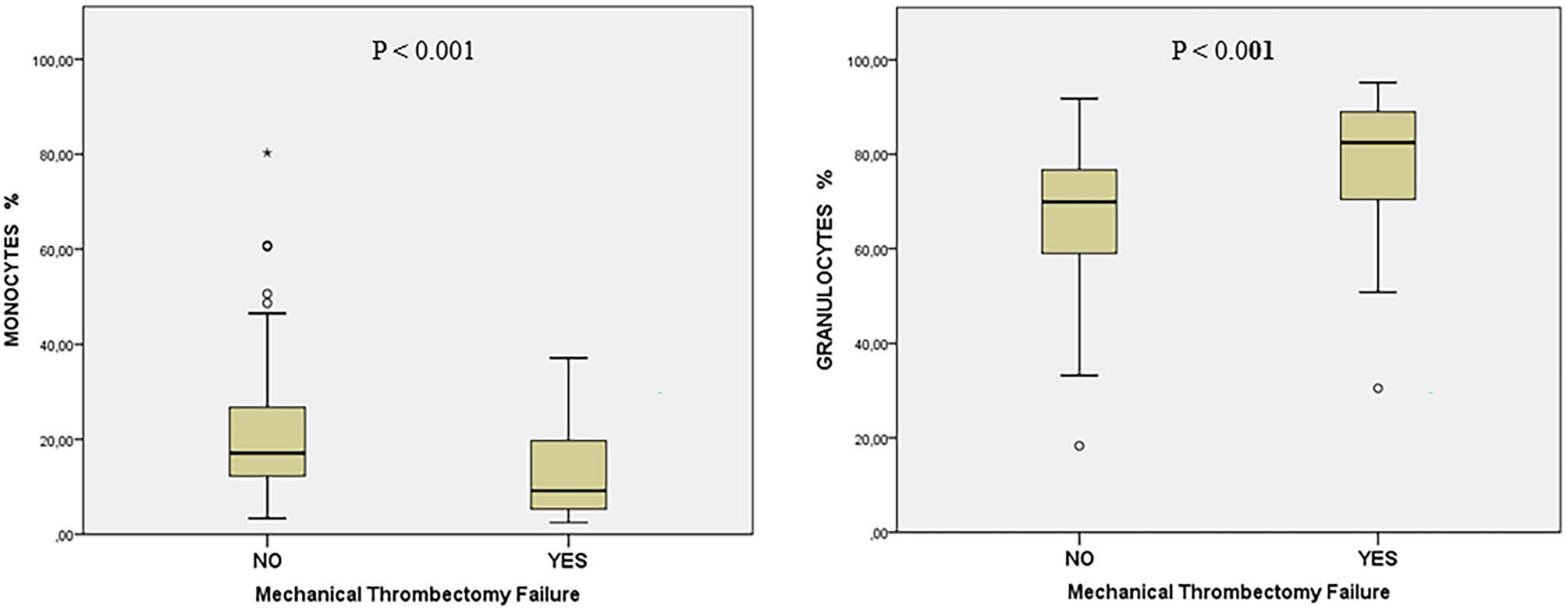
Differences in clot analysis among EVT with multiple passes. MT failure was associated with higher proportion of granulocytes [82.46% (69.19–89.02) vs. 69.90% (59.0–76.84); *p* < 0.001] and lower proportion of monocytes [17.11% (12.18–27.10) vs. 9.18 (5.29–20.23); *p* < 0.001] in clot analysis

In cases that required more than one pass, “successful MT” remained associated with dyslipidemia [47.7% (51) vs. 26.7% (8); *p* = 0.040] and HRCE [63.6% (68) vs. 36.7% (11); *p* = 0.008]. MT failure was associated with higher proportion of granulocytes [82.46% (69.19–89.02) vs. 69.90% (59.0–76.84); *p* < 0.001] and lower proportion of monocytes [17.11% (12.18–27.10) vs. 9.18 (5.29–20.23); *p* < 0.001] in clot analysis (Table 2). Differences in clot composition between groups are represented in Fig. 2.

**Table 2:** EVT with multiple passes: successful MT vs. MT failure

	Total (*n* = 137)	Successful MT(*n* = 107)	MT failure (*n* = 30)	*P*-value
Age, years [IQR]	76 [65–82]	76 [65–82]	77 [63–83]	0.938
Sex (female) *n*, %	73 (53.3)	56 (52.3)	17 (56.7)	0.674
History of atrial fibrillation *n*, %	42 (30.7)	33 (30.8)	9 (30)	0.930
Active smoker *n*, %	28 (20.4)	21 (19.6)	7 (23.3)	0.656
High blood pressure *n*, %	92 (67.2)	72 (67.3)	20 (66.7)	0.949
Diabetes mellitus *n*, %	21 (15.3)	18 (16.8)	3 (10)	0.359
History of coronary artery disease *n*, %	25 (18.2)	22 (20.6)	3 (10)	0.186
Dyslipidemia *n*, %	59 (43.1)	51 (47.7)	8 (26.7)	0.040
Antiplatelets *n* (%)	33 (24.1)	25 (23.4)	8 (26.7)	0.709
Anticoagulation *n* (%)	21 (15.3)	14 (13.1)	7 (23.3)	0.168
Glycemia (mg/dL)	116 [102–133]	118 [102–133]	112 [97–126]	0.329
NIHSS	16 [10–21]	16 [10–21]	16 [8–20]	0.750
HRCE *n* (%)	79 (57.7)	68 (63.6)	11 (36.7)	0.008
LAA *n* (%)	28 (20.4)	18 (16.8)	10 (33.3)	0.071
Other cause *n* (%)	2 (1.5)	1 (0.9)	1 (3.3)	0.333
ASPECTS score [IQR]	9 [8–10]	10 [8–10]	9 [7–10]	0.208
PLVOs	103 (75.1)	80 (74.7)	23 (76.6)	0.832
TICA occlusion *n* (%)	37 (27.0)	28 (26.1)	9 (30)	0.677
Medial artery 1 occlusion *n* (%)	51 (37.2)	38 (35.5)	13 (43.3)	0.435
Medial artery 1 tandem occlusion *n* (%)	10 ( 7.2)	10 (9.3)	0	0.083
BAO occlusion *n* (%)	5 (3.6)	4 (3.7)	1 (3.3)	0.917
MeVOs	34	27 (25.2)	7 (23.3)	0.832
Anterior artery occlusion *n* (%)	3 (2.2)	2 (1.9)	1 (3.3)	0.629
Medial artery 2 occlusion *n* (%)	24 (17.5)	20 (18.6)	4 (13.3)	0.497
Posterior artery occlusion *n* (%)	7 (5.1)	5 (4.7)	2 (6.7)	0.662
iv–rTPA *n* (%)	45 (32.8)	7 (23.3)	7 (23.3)	0.209
Stent retriever first pass, *n* (%)	42 (30.7)	36 (33.6)	6 (20.0)	0.152
Combined, first pass *n* (%)	74 (54.0)	54 (50.5)	20 (66.7)	0.116
ADAPT first pass *n* (%)	20 (14.6)	17 (15.9)	3 (10.0)	0.420
Number of passes [IQR]	3 [2–4]	3 [2–4]	3 [2–5]	0.073
Clot total leukocytes [IQR]	3092 [1283–9765]	3016 [1265–9636]	3679 [1126–11,188]	0.954
Clot granulocytes % [IQR]	71.03 [60.60–81.06]	69.90 [59.0–76.84]	82.46 [69.19–89.02]	< 0.001
Clot monocytes % [IQR]	15.61[10.23–24.40]	17.11 [12.18–27.10]	9.18 [5.29–20.23]	< 0.001
Clot lymphocytes % [IQR]	6.42 [3.01–12.69]	6.63 [3.0–12.80]	5.08 [3.0–12.33]	0.886

*ADAPT* a direct aspiration first pass technique, *EVT* endovascular treatment, *HRCE* high risk cardioembolic etiology, *IQR* interquartile range, *Iv-rTPA* intravenous fibrinolysis, *LAA* large arterial atherosclerosis, *MT* mechanical thrombectomy, *NIHSS* National Institutes of Health Stroke Scale, *PLVOs* proximal large vessel occlusions, *MeVOs* medium vessel occlusions

**Fig. 3:**
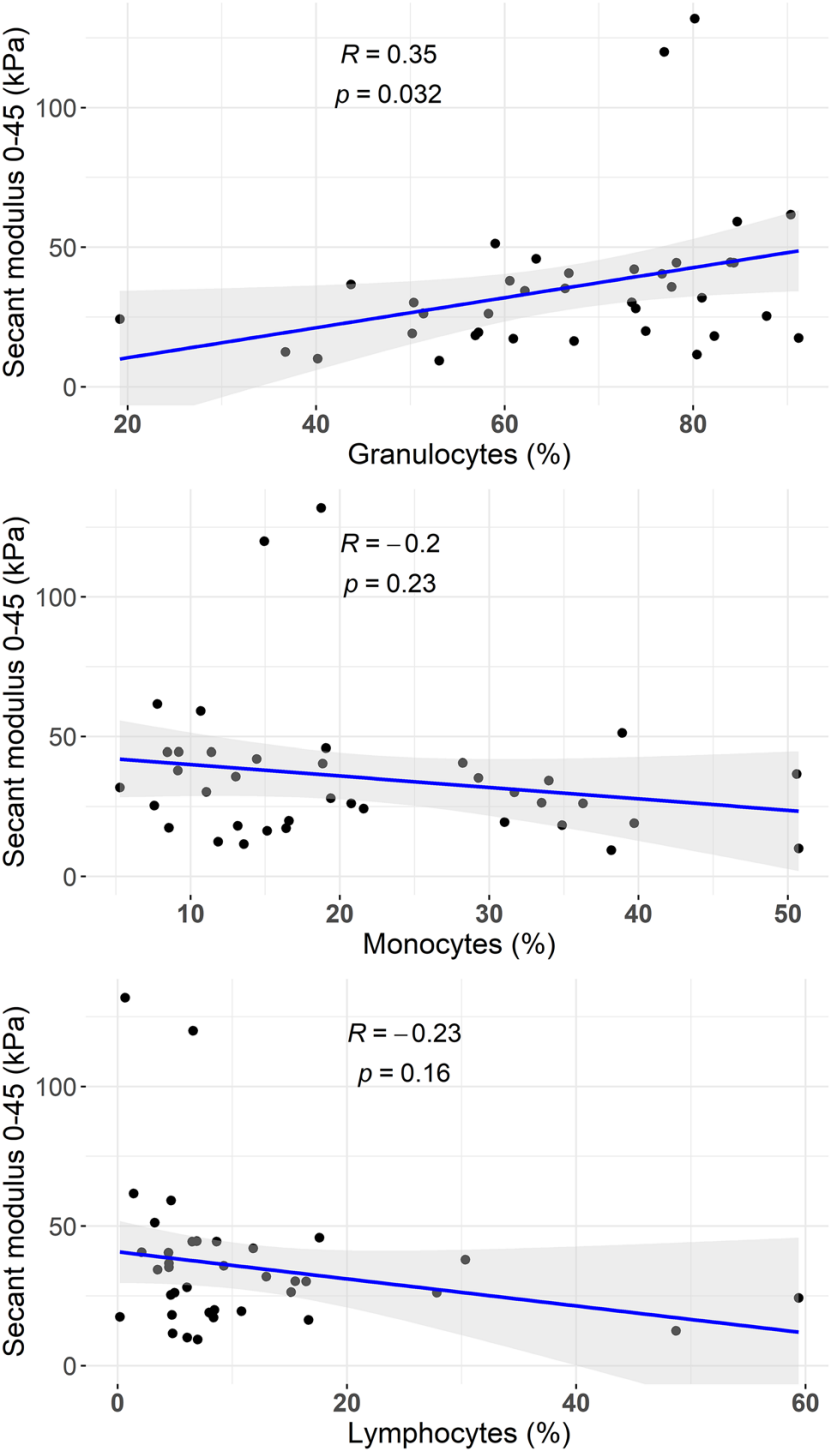
Correlation between clot stiffness and clot composition by flow cytometry

In a multivariable model analysis including significant variables obtained from the univariate analysis (dyslipidemia, HRCE, granulocytes %, and monocytes %), the proportion of granulocytes (aOR 1.07; 95% CI 1.01–1.14) remained as an independent marker of MT failure (Table 3). Youden index’s best cut-off point for clot granulocytes to predict MT failure was 74.40% (AUC of 0.735; sensitivity of 73.33%, specificity of 69.16%, positive predictive value of 40%, negative predictive value of 90.24%, and positive likelihood ratio of 2.38) (Fig. 3). In another multivariate analysis, cut-off point for granulocytes > 74.40% in clot analysis (aOR: 6.99, CI 95%: 2.63–18.59, *p* < 0.001) was independently associated with higher risk of MT failure after adjustment for age, sex dyslipemia, HRCE, and occlusion location (MeVOs). Conversely HRCE decreased the risk of MT failure (aOR 0.234, CI 0.082–0.665, *p* = 0.06), as stated in Table 3.

**Table 3:** Models of multivariate analysis for predictors of MT failure among EVT with multiple passes

	Univariate analysis		Multivariate analysis		
	Unadjusted OR	95% CI	Adjusted OR	95% CI	*P*-value
Dyslipidemia	0.39	0.16–0.97	0.02	0.19–0.85	0.022
HRCE	0.33	0.14–0.76	0.29	0.11–0.76	0.012
Granulocytes %	1.07	1.03–1.11	1.07	1.01–1.14	0.028
Monocytes %	0.925	0.878–0.975	0.99	0.92–1.07	0.914
	Univariate analysis		Multivariate analysis		
	Unadjusted OR	95% CI	Adjusted OR	95% CI	*P*-value
Age	0.99	0.96–1.02	1.01	0.98–1.04	0.341
Sex (female)	1.19	0.52–2.69	1.49	0.560–4.012	0.420
Dyslipidemia	0.39	0.16–0.97	0.36	0.133–0.988	0.047
HRCE	0.33	0.14–0.76	0.24	0.092–0.670	0.006
MeVOS	0.90	0.348–2.336	0.97	0.326–2.921	0.965
Granulocytes > 74.40%	6.16	2.48–15.27	6.99	2.635–18.59	< 0.001

*CI* confidence interval, *HRCE* high risk cardioembolic etiology, *MeVO* medium vessel occlusion, *OR* odds ratio

### Mechanical Characterization and Clot Composition

In another cohort of patients who underwent MT between March and June 2021, thirty-eight clot fragments were analyzed from samples obtained from twenty-one MTs. In this sub-analysis, unconfined compression tests were conducted before flow cytometry. Clinical characteristics of all patients and experimental data from mechanical testing and flow cytometry analysis are summarized in Online Resource 2. Median age was 80 [72–88] and median clot stiffness was 30.2 (IQR, 18.9–42.7) kPa. Although no significant relationship was found between monocytes (*r* = − 0.23, *p* = 0.177), lymphocytes (*r* = − 0.187, *p* = 0.274), and clot stiffness, there was a positive correlation between granulocyte proportion and thrombi stiffness (Pearson’s *r* = 0.35, *p* = 0.032), as shown in Fig. 3.

## Discussion

Based on our results, high proportion of granulocytes of intracranial clots is a marker of MT failure during standard acute EVT of LVO strokes. Most published studies have employed anatomo-pathology techniques to evaluate the influence of thrombi composition and MT outcome [24]. It has been reported that platelet-rich clots are difficult to retrieve with poorer revascularization outcomes; there is a positive correlation between platelets and white blood cells, suggesting a possible inflammatory pathway for a pro-coagulant mechanism [25]. Intracranial clots with high proportion of leukocytes promote thrombi formation by extracellular DNA traps [9]. Web-like structures called NETs are released from activated neutrophils, which create a scaffold for thrombus formation. The amount of leukocytes and NETs may increase the adherence to the vessel wall, making the removal of thrombus more difficult. At the same time, neutrophils are involved in neuronal cell death [26]. Flow cytometry studies showed that neutrophils are triggered by ischemia in experimental models of direct occlusion in the absence of reperfusion [27], and a recent study in humans observed leukocyte invasion distal to intracranial LVO primarily attributable to strong elevation of the neutrophil subpopulation [28].

Global analysis of stroke etiology and EVT outcomes showed a significant association between LAA strokes and MT failure. However, this was not confirmed in the sub-analysis in EVT with multiple passes.

Our study evidences the relationship between granulocyte-rich clots and increased thrombus stiffness. Previous studies have shown that fibrin/platelet-rich thrombi are stiffer than erythrocyte-rich clots and present higher frictional properties that might contribute to resistance to MT. On another note, erythrocyte-rich clots might be more prone to fracture and present higher risk of per procedural embolization since the primary mechanism of clot deformation is fibrin rupture [21, 22, 29, 30].

Clot stiffness quantified in our study matched previously reported values on erythrocyte-rich (secant modulus [0–45%] = 26 ± 2 kPa) and fibrin-rich (secant modulus [0–45%] = 170 ± 39 kPa) thrombi harvested from clinical cases [22]. Similar clot stiffness values using the unconfined compression experiments (tangent modulus at 40% strain = 59 ± 63 kPa) has been published and found no association between total leukocytes and thrombus stiffness [21]. However, our study focused on specific leukocyte subpopulations achieving a positive correlation between granulocyte proportion and thrombi stiffness, which may explain why high granulocyte content within the clot of LVO leads to resistance to recanalization with standard MT procedures.

Our protocol of FC that analyzed intracranial clots might help to identify thrombectomy-resistant thrombus in acute stroke treatment. FC technique allowed to perform a fast pathway of clot analysis, avoiding time-consuming steps of standard anatomo pathologic techniques. To our knowledge, no previous studies have employed FC to study clot composition in cases of MT failure.

First goal of MT is to achieve first pass recanalization (TICI 3 after first pass) with conventional techniques (stent retriever, direct aspiration, or combination of these techniques) within fast procedures [1] but there are no evidence of which should be the best strategy after first attempt failure. Besides sudden recanalization strongly predicts favorable outcome in patients undergoing EVT even after previous unsuccessful attempt, higher number of passes are associated with worse outcome probably due to vessel harm during mechanical thrombectomy, clot fragmentations and higher risk of clinical complications [7]. Obtaining a marker after first pass that predict high risk of mechanical thrombectomy failure with conventional MT techniques may help to select patients for advanced procedures such us permanent intracranial stenting (PIS) as a rescue reperfusion technique in order to avoid complications and procedure delay [31].

On the other hand, because neutrophils and NETs are important constituents of cerebral thrombi, there are currently some groups trying to develop new thrombolytic agents for targeting NETs with DNase 1 that might have potential acute stroke treatment in combination with endovascular treatment [32].

There are new sensors designed to discriminate different types of thrombus during EVT, but no device has been demonstrated yet to improve the decision-making process during EVT of acute stroke treatment [33]. Future works might focus on the development of a simplified FC analysis that might analyze part of the intracranial clot during MT by a point-of-care test might be worthy as a guide for patient-specific treatment strategy. Moreover, intraarterial blood testing [28] around the clot might potentially offer promising results without the need for the analysis of the clot itself, but this hypothesis should be tested in further studies.

This study has several limitations. Clots were not obtained in every procedure after catheter removal. In fact, in some MT failure cases, no thrombus is collected after many passes. The small cohort of patients included in the second study makes the results less robust compared to the global results of the first analysis. Samples stored for 24–48 h may be used for immunophenotyping, but time spent in mechanical characterization of clots may provoke losses in absolute cell numbers in FC analysis; these limitations should be taken into account for future analysis [19]. We did not employ a specific marker of neutrophils in our analysis, and we assumed that higher percentage of granulocytes was due to higher neutrophils and potentially higher NETS. Dyslipidemia was associated with successful recanalization in our study, but our data was only based on medical records and not on cholesterol levels in blood tests. Future studies to explore the interaction between granulocytes and platelets in intracranial thrombi and their impact on endovascular treatment are warranted. Despite registering the type of device employed, we did not analyze its influence on clot composition variation, so future work should evaluate the physical properties of the clot according to FC composition to design next-generation EVT devices [21, 34, 35].

## Conclusions

There is a positive correlation between granulocyte proportion and thrombi stiffness that may explain endovascular resistance to recanalization. Influence of granulocytes within thrombus may be a target for future reperfusion treatments.

## Supplementary Information

Below is the link to the electronic supplementary material.

Supplementary file1 (DOCX 504 kb)

Supplementary file2 (DOCX 39 kb)

## Data Availability

The data that support the findings of this study are available on request from the corresponding author.

## References

[1] Zaidat O, Castonguay A, Linfante I (2018). First pass effect a new measure for stroke thrombectomy. Stroke.

[2] Jindal G, Carvalho HP, Wessell A (2019). Beyond the first pass: revascularization remains critical in stroke thrombectomy. J Neurointerv Surg.

[3] Tonetti DA, Desai SM, Casillo S (2020). Successful reperfusion, rather than number of passes, predicts clinical outcome after mechanical thrombectomy. J Neurointerv Surg.

[4] Garcia-Tornel A, Rubiera M, Requena M (2020). Sudden recanalization: a game-changing factor in endovascular treatment of large vessel occlusion strokes. Stroke.

[5] Premat K, Dechartres A, Lenck S (2020). Rescue stenting versus medical care alone in refractory large vessel occlusions: a systematic review and meta-analysis. Neuroradiology.

[6] Ribo M, Flores A, Rubiera M (2013). Difficult catheter access to the occluded vessel during endovascular treatment of acute ischemic stroke is associated with worse clinical outcome. J Neurointerv Surg.

[7] Garcia-Tornel A, Requena M, Rubiera M (2019). When to stop. Stroke.

[8] Maekawa K, Shibata M, Nakajima H (2018). Erythrocyte-rich thrombus is associated with reduced number of maneuvers and procedure time in patients with acute ischemic stroke undergoing mechanical thrombectomy. Cerebrovasc Dis Extra.

[9] Heo JH, Nam HS, Kim YD (2020). Pathophysiologic and therapeutic perspectives based on thrombus histology in stroke. J Stroke.

[10] Stracke CP, Fiehler J, Meyer L (2020). Emergency intracranial stenting in acute stroke: predictors for poor outcome and for complications. J Am Heart Assoc.

[11] Adams HP, Bendixen BH, Kappelle LJ, Marsh EE (1993). Classification of subtype of acute ischemic stroke. Definitions for use in a multicenter clinical trial. Toast. Trial of org 10172 in acute stroke treatment. Stroke.

[12] Ribo M, Boned S, Rubiera M (2017). Direct transfer to angiosuite to reduce door-to-puncture time in thrombectomy for acute stroke. J Neurointerv Surg.

[13] Powers WJ, Rabinstein AA, Ackerson T (2019). Guidelines for the early management of patients with acute ischemic stroke: 2019 update to the 2018 guidelines for the early management of acute ischemic stroke: a guideline for healthcare professionals from the American Heart Association/American Stroke Association. Stroke.

[14] . Saver JL, Chapot R, Agid R, Hassan A, Jadhav AP, Liebeskind DS, et al. Thrombectomy for distal, medium vessel occlusions: a consensus statement on present knowledge and promising directions. Stroke. 2020:STROKEAHA120028956. 10.1161/STROKEAHA.120.028956 32757757

[15] Ospel J, Goyal M (2021). A review of endovascular treatment for medium vessel occlusion stroke. J Neurointerv Surg..

[16] Munich SA, Vakharia K, Levy EI (2019). Overview of mechanical thrombectomy techniques. Neurosurgery.

[17] Almekhlafi MA, Mishra S, Desai JA (2014). Not all “successful” angiographic reperfusion patients are an equal validation of a modified tici scoring system. Interv Neuroradiol.

[18] Davis BH, Dasgupta A, Kussick S (2013). Validation of cell- based fluorescence assays: practice guidelines from the ICSH and ICCS –part II – preanalytical issues. Cytometry Part B.

[19] Diksa AM, Bonroyb C, Teodosioa C, Groenlanda RJ, de Mooija B, de Maertelaerec E (2019). Impact of blood storage and sample handling on quality of high dimensional flow cytometric data in multicenter clinical research. J Immunol Methods.

[20] Juega J, Palacio-Garcia C, Rodriguez M, et al. Monocyte-to-lymphocyte ratio in clot analysis as a marker of cardioembolic stroke etiology. Transl Stroke Res. 2021. 10.1007/s12975-021-00946-w 34586594

[21] Boodt N, Snouckaert van Schauburg PRW, Hund HM (2021). Mechanical characterization of thrombi retrieved with endovascular thrombectomy in patients with acute ischemic stroke. Stroke.

[22] Chueh JY, Wakhloo AK, Hendricks GH (2011). Mechanical characterization of thromboemboli in acute ischemic stroke and laboratory embolus analogs. AJNR Am J Neuroradiol.

[23] Malone F, McCarthy E, Delassus P (2018). The mechanical characterisation of bovine embolus analogues under various loading conditions. Cardiovasc Eng Technol.

[24] Staessens S, Denorme F, Francois O (2020). Structural analysis of ischemic stroke thrombi: histological indications for therapy resistance. Haematologica.

[25] Douglas A, Fitzgerald S, Mereuta OM (2020). Platelet-rich emboli are associated with von Willebrand factor levels and have poorer revascularization outcomes. J Neurointerv Surg..

[26] Laridan E, Denorme F, Desender L (2017). Neutrophil extracellular traps in ischemic stroke thrombi. Ann Neurol.

[27] Perez-de-Puig I, Miro-Mur F, Ferrer-Ferrer M (2015). Neutrophil recruitment to the brain in mouse and human ischemic stroke. Acta Neuropathol.

[28] Kollikowski AM, Schuhmann MK, Nieswandt B, et al. Local leukocyte invasion during hyperacute human ischemic stroke. Ann Neurol. 2020 Mar;87(3):466–479. 10.1002/ana.25665 31899551

[29] Weafer FM, Duffy S, Machado I (2019). Characterization of strut indentation during mechanical thrombectomy in acute ischemic stroke clot analogs. J Neurointerv Surg.

[30] Gunning GM, McArdle K (2018). Clot friction variation with fibrin content; implications for resistance to thrombectomy. J Neurointerv Surg..

[31] Perez-Garcia C, Gomez-Escalonilla C, Rosati S (2020). Use of intracranial stent as rescue therapy after mechanical thrombectomy failure-9-year experience in a comprehensive stroke centre. Neuroradiology.

[32] Chen R, Zhang X, Gu L (2021). New insight into neutrophils: a potential therapeutic target for cerebral ischemia. Front Immunol.

[33] Messina P, Garcia C, Rambeau J (2022). Impedance-based sensors discriminate among different types of blood thrombi with very high specificity and sensitivity. J Neurointerv Surg.

[34] Li J, Castano O, Tomasello A, et al. Catheter tip distensibility substantially influences the aspiration force of thrombectomy devices. J Neurointerv Surg. 2022;14(1). 10.1136/neurintsurg-2021-017487 33858973

[35] Staessens S, Francois O, Brinjikji W (2021). Studying stroke thrombus composition after thrombectomy: what can we learn?. Stroke.

