# Supplementary material for: Granulocytes-Rich Thrombi in Cerebral Large Vessel Occlusion Are Associated with Increased Stiffness and Poorer Revascularization Outcomes
Source: Neurotherapeutics. 2023 May 22;20(4):1167–76. doi: 10.1007/s13311-023-01385-1 (PMC10457261; doi:10.1007/s13311-023-01385-1)
Supplement: Supplementary file 1 — Supplementary file1 (DOCX 504 kb) [file 13311_2023_1385_MOESM1_ESM.docx]

**Supplemental information**

**Figure 1. Compression tests**

**Type:** non-destructive test procedure to determine resistance to compression.

Procedure:

1. Cover the compression plates with paraffin film.

2. Place the specimen on the lower platform as shown in Figure 4(c).

3. Using the software: set a maximum strain of 10 % at 0.25 mm/s, do not define any specific specimen geometry. Start the test.

**Type:** non-destructive test procedure to determine **resistance to compression.**


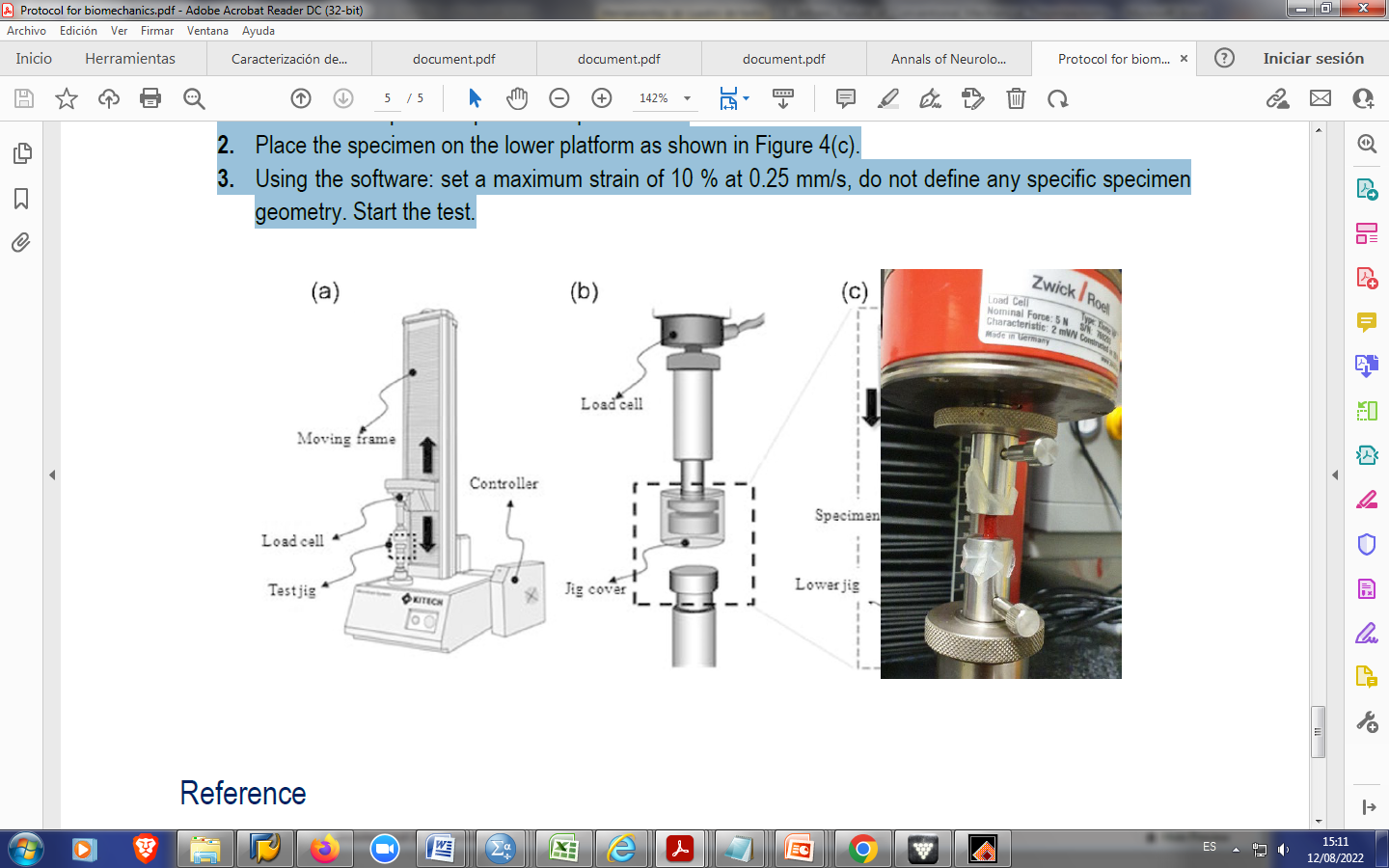


| **Patient**  **Table 1. Clinical characteristics, clot stiffness from unconfined compression tests, and clot composition determined by flow cytometry analysis.** | **Age** | **Sex** | **Occlusion location** | **iv fibrinolysis** | **MT Technique** | **N passes** | **Pass-Sample** | **Stiffness [0-45%] (kPa)** | **Lymphocytes (%)** | **Monocytes (%)** | **Granulocytes (%)** | **Final mTICI** |
| --- | --- | --- | --- | --- | --- | --- | --- | --- | --- | --- | --- | --- |
| 1 | 93 | F | ICA-T | YES | Combined | 1 | 1-A | 35.3 | 4.47 | 29.27 | 66.40 | 3 |
|  |  |  |  |  |  |  | 1-B | 59.2 | 4.66 | 10.68 | 84.66 |  |
| 2 | 89 | F | ICA-T | NO | Combined | 1 | 1-A | 45.9 | 17.60 | 19.07 | 63.33 | 2c |
|  |  |  |  |  |  |  | 1-B | 26.2 | 27.84 | 20.76 | 51.40 |  |
|  |  |  |  |  |  |  | 1-C | 26.4 | 15.13 | 33.50 | 51.37 |  |
| 3 | 80 | M | Proximal ICA | YES | Combined | 3 | 1-A | 38.0 | 30.32 | 9.14 | 60.54 | 2b |
|  |  |  |  |  |  |  | 1-B | 30.3 | 15.49 | 11.05 | 73.48 |  |
|  |  |  |  |  |  |  | 1-C | 25.4 | 4.64 | 7.57 | 87.79 |  |
|  |  |  |  |  |  |  | 1-D | 42.1 | 11.83 | 14.43 | 73.73 |  |
| 4 | 77 | F | Proximal ICA | NO | Combined | 1 | 1-A | 35.8 | 9.26 | 13.03 | 77.7 | 2c |
|  |  |  |  |  |  |  | 1-B | 20.0 | 8.45 | 16.59 | 74.96 |  |
| 5 | 69 | M | ICA-T | YES | Combined | 8 | 4-A | 24.3 | 59.39 | 21.59 | 19.17 | 3 |
|  |  |  |  |  |  |  | 4-B | 18.4 | 8.24 | 34.88 | 56.89 |  |
|  |  |  |  |  |  |  | 8-A | 40.5 | 4.43 | 18.84 | 76.70 |  |
|  |  |  |  |  |  |  | 8-B | 51.3 | 3.23 | 38.90 | 59.00 |  |
| 6 | 77 | M | P1 | YES | Combined | 3 | 1-A | 18.2 | 4.73 | 13.15 | 82.24 | 3 |
| 7 | 80 | F | M1 | NO | Combined | 1 | 1-A | 44.6 | 6.91 | 9.20 | 83.95 | 2c |
| 8 | 81 | F | M1 | NO | Combined | 2 | 2-A | 19.5 | 10.79 | 31.02 | 57.22 | 2b |
| 9 | 70 | M | M1 | YES | Combined | 1 | 1-A | 30.2 | 16.43 | 31.68 | 50.34 | 2c |
| 10 | 84 | M | ICA-T | NO | Combined | 3 | 1-A | 17.5 | 0.19 | 8.55 | 91.19 | 2c |
| 11 | 96 | F | ICA-T | NO | Combined | 4 | 1-A | 31.9 | 12.96 | 5.26 | 80.92 | 0 |
|  |  |  |  |  |  |  | 2-A | 12.5 | 48.69 | 11.85 | 36.74 |  |
| 12 | 90 | M | ICA-T | NO | Combined | 1 | 1-A | 19.1 | 7.98 | 39.70 | 50.20 | 3 |
|  |  |  |  |  |  |  | 1-B | 17.3 | 8.36 | 16.40 | 60.90 |  |
| 13 | 65 | F | ICA-T | YES | Combined | 3 | 1-A | 120.0 | 6.58 | 14.94 | 76.92 | 1 |
|  |  |  |  |  |  |  | 1-B | 44.5 | 8.63 | 11.39 | 78.21 |  |
|  |  |  |  |  |  |  | 1-C | 61.7 | 1.39 | 7.77 | 90.35 |  |
| 14 | 76 | M | BA | YES | Aspiration | 3 | 1-A | 36.7 | 4.47 | 50.58 | 43.70 | 2c-3 |
|  |  |  |  |  |  |  | 1-B | 10.1 | 6.07 | 50.72 | 40.17 |  |
| 15 | 66 | M | M1 | NO | Combined | 3 | 1-A | 11.6 | 4.80 | 13.56 | 80.39 | 2c |
| 16 | 101 | F | M1 | YES | Combined | 1 | 1-A | 9.4 | 6.98 | 38.17 | 53.06 | 2c |
| 17 | 77 | M | M1 | YES | Combined | 2 | 2-A | 40.7 | 2.10 | 28.23 | 66.81 | 3 |
| 18 | 85 | M | ICA-T | YES | Combined | 1 | 1-A | 34.4 | 3.48 | 33.99 | 62.13 | 2c |
|  |  |  |  |  |  |  | 1-B | 26.2 | 4.97 | 36.27 | 58.28 |  |
| 19 | 86 | F | M2 | NO | Combined | 1 | 1-A | 16.4 | 16.65 | 15.12 | 67.37 | 3 |
| 20 | 75 | F | M1 | NO | Combined | 1 | 1-A | 28.1 | 6.04 | 19.38 | 73.90 | 2b |
|  |  |  |  |  |  |  | 1-B | 44.5 | 6.51 | 8.44 | 84.33 |  |
| 21 | 56 | M | M2 | NO | Combined | 2 | 1-A | 131.9 | 0.65 | 18.75 | 80.17 | 2c |
